# Postcoital vaginoperitoneal fistula after hysterectomy for gynecological malignancy

**DOI:** 10.4103/0974-2700.66547

**Published:** 2010

**Authors:** Shilpa Singla, Sunesh Kumar Jain

**Affiliations:** Department of Obstetrics and Gynecology, All India Institute of Medical Sciences, New Delhi – 110 029, India

**Keywords:** Pneumoperitoneum, postcoital fistula, vaginoperitoneal fistula

## Abstract

A true incidence of postcoital fistula is not known as it is seldom suspected. It presents as acute pneumoperitoneum with signs and symptoms same as that of perforation of a hollow viscus. A 38-year-old parous woman presented with postcoital fistula 10 weeks after panhysterectomy for carcinoma ovary stage IIIc. Pneumoperitoneum was detected, with large amount of gas under the diaphragm on radiograph of the abdomen. A breach was found in the vaginal vault. All other causes of fistula were excluded. Fistula healed spontaneously on follow-up.

## INTRODUCTION

The most common causes of pneumoperitoneum are iatrogenic: postoperatively after laparotomy or laparoscopy. These findings usually resolve by 1 week postoperatively. Nonsurgical pneumoperitoneum accounts for approximately 10% of all the cases.[1] Nonsurgical causes of pneumoperitoneum may be due to positive pressure ventilation, pneumatosis cystoids intestinalis, or entry through female genital tract. Injury, foreign body, vigorous vaginal douching, and sexual trauma are the other rare causes of letting air in through the female genitalia. Sexual trauma is a very rare cause of vaginal vault fistulas.[2]

We report a rare case of vaginal vault fistula following coitus, following a hysterectomy done for malignancy.

## CASE REPORT

A 38-year-old parous woman, presented to outpatient gynecology department with complaints of abdominal distension and bloating for the last 2 months. On clinical examination, ascites was present. On per vaginum examination, bilateral adnexal masses were present; vagina was healthy. There was absence of nodularity or deposits in the pouch of Douglas. Ultrasonographic evaluation revealed 4 × 4 cm complex adnexal cyst on the left side, with the presence of ascites. Ascitic fluid examination revealed malignant cells following which a decision was taken for laparotomy. Laparotomy was performed with panhysterectomy, bilateral pelvic lymphadenectomy, and infracolic omentectomy. Peroperatively, 800 mL straw colored ascites was present. Right ovarian 5 × 5 cm and left ovarian 4 × 4 cm masses were present with the presence of variegated consistency and necrotic areas on cut sections. Omental caking was present. Histopathologic diagnosis of papillary serous cystadenocarcinoma of ovary, stage IIIc was confirmed. There was no evidence of metastasis to vagina. She was discharged in a stable condition with advised abstinence for 6 weeks to allow vaginal vault healing. Postoperatively, 6 cycles of chemotherapy with paclitaxel and carboplatin, at 3 weekly intervals was planned.

**Figure 1:**
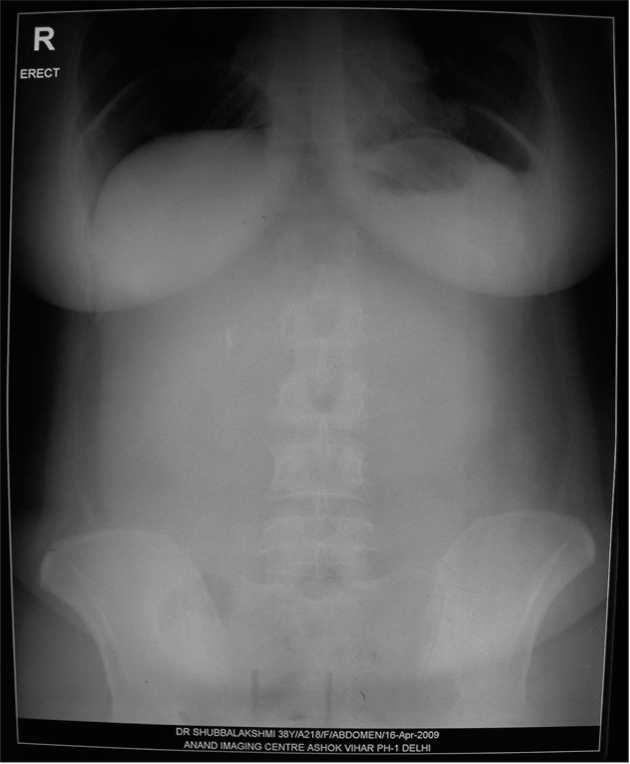
Radiograph of the abdomen (erect) showing free gas under diaphragm

She presented in gynecologic emergency 10 weeks after her hysterectomy (after her third cycle of chemotherapy) with acute abdominal pain. There was no associated fever or bowel or bladder symptom. She had been tolerating her meals well. There was a history of coital act, the night before. This was her first coitus after surgery. Her general physical examination was unremarkable. Her pulse rate was 86/min, respiratory rate was 18/min, blood pressure was 120/80 mmHg. She was afebrile; oral temperature was 99F. On local per speculum examination, 1 cm rent negotiating a finger tip was discovered at the vaginal vault. There was no active bleeding or signs of inflammation. Upright chest radiograph revealed gas under diaphragm indicating pneumoperitoneum [Figure 1]. Ultrasound examination of the abdomen showed no significant pelvic mass. Rest of the abdomen was also unremarkable. A repeat chest radiography and erect abdominal radiography, done one day later revealed same gas under diaphragm.

Surgical opinion was taken to rule out acute intestinal perforation. In view of absence of clinical bowel symptoms, perforation seemed unlikely, so barium meal test and barium enema were not performed. There was no history of any foreign body insertion, vigorous douching, fall, or any other trauma. Iatrogenic persistence of air in the peritoneum is unlikely to persist 10 weeks postsurgery. On clinical examination, there was no evidence of fresh tumor deposits or nodularity in the pouch of Douglas, which might have caused fistula as a progression of malignancy. In 10 weeks, the vaginal vault is expected to heal. The patient was asymptomatic till she had coitus allowing the authors to assume there was no defect in healing. In the face of other negative results, onset of pain after coitus, and presence of rent in the vault, a diagnosis of vaginal vault fistula, postcoitus was made.

**Figure 2:**
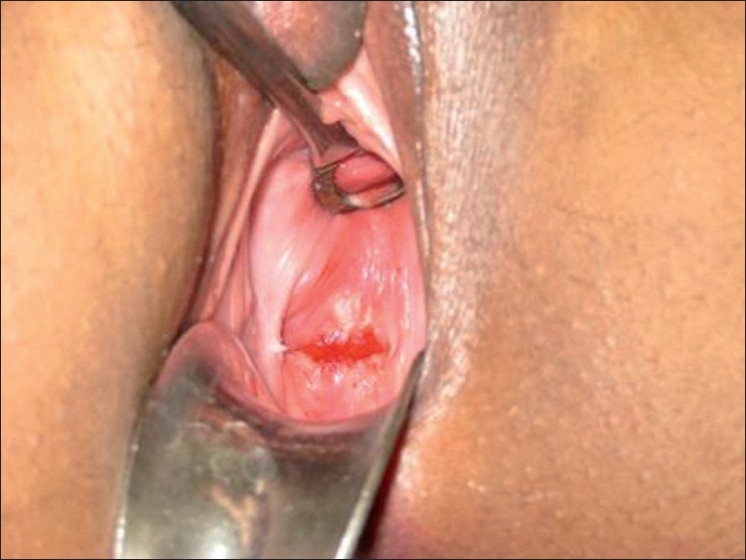
Per speculum examination of vaginal vault showing healed ulcer at 2-week follow-up

She was administered antibiotics, analgesics, and asked to keep abstinence. She was told to avoid any vaginal douching or undergo any intervention. Pain gradually subsided over the next 3 days. Because the size of the fistula was small, it was decided not to actively intervene. Per speculum examination after 1 week revealed healed ulcer at the vaginal vault [Figure 2], and a follow-up examination after 2 weeks revealed an intact vault.

## DISCUSSION

In most cases, such acute abdomen and pneumoperitoneum present to the surgeon, and perforation of a hollow viscus is the underlying cause.[3] The first reference to the female genital tract as a possible conduit for peritoneal air was found in the French literature, due to high pressure vaginal douching.[4] First case of pneumoperitoneum due to postcoital fistula after abdominal hysterectomy was reported in 1980.[5]

Vesicovaginal fistula and pneumperitoneum due to rough coital activity and vigorous intercourse have usually been reported in young nullipara status females.[2,5,6] Postpartum and postmenstrual state may be relatively prone periods to produce flatus vaginalis leading to pneumperitoneum.[6] Early resumption of sexual act in postpartum period and even knee–chest exercises may induce air in peritoneum. Hysterectomy is an other potential high-risk condition, which may predispose to peritoneal–vaginal fistula. This may be due to persistent defective tracts following surgery at the vault. Secondly, acute vaginal laceration due to coitus, on the already weakened tissues may be a contributing factor.[7]

There has been no such case report of postcoital fistula after hysterectomy done for malignant female genital disease. With presentation of our case, we further hypothesize that in hysterectomy done for malignant indication, the vaginal vault might be relatively weaker than hysterectomy done for benign diseases. This may be due to low immunity status and poor healing, making these women more prone to such sexual fistulae.

Surgical intervention might not be needed in all cases. If there is any suspicion of perforated viscus, diagnostic laparoscopy and laparotomy to explore and repair the bowel should be performed. If the patient’s examination is negative for the signs of surgical abdomen, “wait and watch” policy may be adopted. Cases with resolution of symptoms may be followed-up. The cases with worsening of symptoms or with persistent or new appearance of signs of peritonitis or inflammation may be considered for surgical intervention.[8] In peritoneovaginal fistula, the patient might appear acutely distressed due to large amount of free air under diaphragm but objective signs, such as fever, tachycardia, hypotension, and leukocytosis are absent. Unlike gastrointestinal or urinary tract fistulae, there is no constant stream of dribbling material through these fistulae, which might keep it patent, so they are likely to resolve spontaneously. The patient should be advised against temporary measures, such as intercourse, douching, or any activity that might force air or liquid through the fistulous tract under pressure. Surgical option might be considered if fistula does not heal spontaneously after a long time, persists, or gets secondarily infected.[7]

## CONCLUSION

While assessing a woman with acute abdomen with pneumoperitoneum in emergency, it is necessary to have a high index of suspicion and consider all etiologies, including sexual trauma. In surgical specialties, a history of woman is not complete without gynecologic history, which should include detailed menstrual and sexual history. Coital activity should be considered in the differential diagnosis of pneumoperitoneum. This can help to avoid unnecessary negative laparotomy and late diagnosis of coital fistula.[9,10] Decision for surgical intervention can be deferred by judicial assessment of the case.
